# The state of the ‘GMO’ debate - toward an increasingly favorable and less polarized media conversation on ag-biotech?

**DOI:** 10.1080/21645698.2022.2051243

**Published:** 2022-03-23

**Authors:** Sarah Evanega, Joan Conrow, Jordan Adams, Mark Lynas

**Affiliations:** a The Alliance for Science, the Boyce Thompson Institute, Ithaca, New York, USA; b Cision Global Insights, Ann Arbor, Michigan, USA

**Keywords:** GMO, agricultural biotechnology, biotechnology, media coverage, social media, genetic engineering, sentiment analysis

## Abstract

Although nearly three decades have passed since genetically modified crops (so-called ‘GMOs’) were widely commercialized, vociferous debate remains about the use of biotechnology in agriculture, despite a worldwide scientific consensus on their safety and utility. This study analyzes the volume and tenor of the GMO conversation as it played out on social and traditional media between 2018 and 2020, looking at 103,084 online and print articles published in English-language media around the world as well as 1,716,071 social media posts. To our knowledge, our analysis is the first comprehensive survey of the shifting traditional and online media discourse on this issue during this time period. While the volume of traditional media coverage of GMOs increased significantly during the period, this was combined with a dramatic drop in the volume of social media posts of over 80%. Traditional media tended to be somewhat more positive in their coverage than social media in 2018 and 2019, but that gap disappeared in 2020. Both traditional and social media saw trends toward increasing favorability, with the positive trend especially robust in social media. The large decline in volume of social media posts, combined with a strong trend toward greater favorability, may indicate a drop in the salience of the GMO debate among the wider population even while the volume of coverage in traditional media increased. Overall, our results suggest that both social and traditional media may be moving toward a more favorable and less polarized conversation on ag-biotech overall.

## Introduction

Major international and national expert institutions and academies accept the scientific consensus that food produced from genetically modified (GM) crops is as safe as any other, and that no specific safety risks or health concerns can be attributed to consumption of so-called GMOs.^1,2^ However, public opinion across the world has been markedly skeptical of GMOs since they were first introduced into the food supply in 1994. Some of the most frequently cited concerns are fears about food safety, corporate control of seeds and the food supply, potential pesticide use associated with the crops, and the welfare of smallholder farmers.

In China, for example, a survey carried out in 2016 found that 47% of people held a negative view of GMOs, with nearly 14% believing that “GM technology was a form of bioterrorism targeted at China.”^3^ In Kenya, where the government initiated a ban on GM imports in 2013 but has recently permitted farmers to begin growing GM cotton, about a third of those polled held a negative opinion of GMOs as long ago as 2003.^4^ In some European countries, opposition to GMOs can be particularly high: in Poland, a 2016 survey found that over 60% of respondents opposed the production and distribution of GM foods in the country.^5^

This public suspicion is not shared by most scientists. A Pew Research Center survey conducted in the United States in 2015 detected a wider gap between scientists and the public on attitudes toward GMOs than any other area of science-related controversy, including vaccines, nuclear power, and pesticides. Specifically, only 37% of the general public thought that GM foods were safe to eat, compared to 88% of AAAS scientists.^6^ Pew also found in 2016 that the US public was almost entirely unaware of the high level of consensus on GMO safety that exists in the scientific community, with only 14% of people concurring that “almost all of scientists agree that GM foods are safe to eat.”^7^

Newer studies indicate more favorable public sentiment toward GM products. These include a study by the European Food Safety Authority that saw the percentage of Europeans choosing GMOs as a food safety concern drop from 66% in 2010 to just 27% in 2019^8^ and an October 2019 Pew poll that found a majority of Americans surveyed believe it is likely that GM crops will increase the global food supply and result in more affordable food prices.^9^

This study seeks to evaluate the volume, reach, and sentiment of the social and traditional media conversation around GMOs over a three-year period between January 2018 and December 2020. It aims to shed light on such questions as how media coverage may influence public perceptions, whether media share scientific perceptions around GMOs, how traditional and social media cover the issue, the influence of certain companies in affecting the tone of the conversation, the role of bots and cyborgs in the conversation, how the volume of coverage has shifted, and attitudes toward emerging tools in agricultural biotechnology.

## Methods

Source data was gathered by Cision Media Insights, which combined 200 pre-defined top tier English-language media and 75,000 online media with social media to analyze trends in the GMO debate globally. Based on media availability, content is sourced via an in-house clipping service, automated feeds based on keywords (third-party API), manual searches for online content behind paywalls and database-sourced print media. Social media coverage includes English-language Twitter feeds and public Facebook pages. Content was captured using relevant keywords (See Supplementary Information for a list of top-tier media and keywords).

This content was subjected to automated computer analysis in real time, using Cision’s natural language processing and custom dictionaries, including a black/white list to help eliminate irrelevant content. Human analysis was included for relevance and sentiment validation of 10,800 top-tier English language articles and 54,000 social media posts, with analysis of the remainder being automated. In total 103,084 traditional media articles covering GMOs were analyzed, alongside 1,716,071 pieces of social media content.

For sentiment analysis, content was assigned a ‘positive’ tag if the statement generally would likely leave the reader feeling more positive about the corporations, individuals, or issues mentioned or if the journalist took a positive stance. A ‘negative’ tag was assigned if a statement would leave the reader likely feeling more critical or if the journalist took a negative stance. Factual explanations of the benefits of biotechnology would count as ‘positive,’ for example, while critiques would count as ‘negative.’ A neutral statement would express no position and the reader would likely not be swayed in any direction. The overall favorability value combines ‘positive’ and ‘neutral’ sentiment into a single value. We also use the ‘mixed’ or ‘ambivalent’ sentiment designation for lines of text that contain a positive and negative element. For an example, a statement such as “while studies have shown that GMO foods are safe to eat, or even safer than organic foods, their relationship to pesticides is a dangerous concern.” Full details of the Cision sentiment analysis method are given in Supplementary Information.

We use the term ‘gross reach’ to indicate the total potential audience of a media item, meaning the number of people who might have had the opportunity to see an original article or social media post, including reposts, replies, and retweets/shares of a social post. For print this includes the number of printed copies of a publication multiplied by the average number of readers per copy. For online this includes monthly page impressions of the URL of the given outlet (including sub-page impressions separately where possible) divided by the average number of published articles for that outlet. These readership and page impression counts for print and online are provided by third parties such as Nielsen. For social media, reach is based on the number of followers of the social media account.

## Results

**Figure 1.**
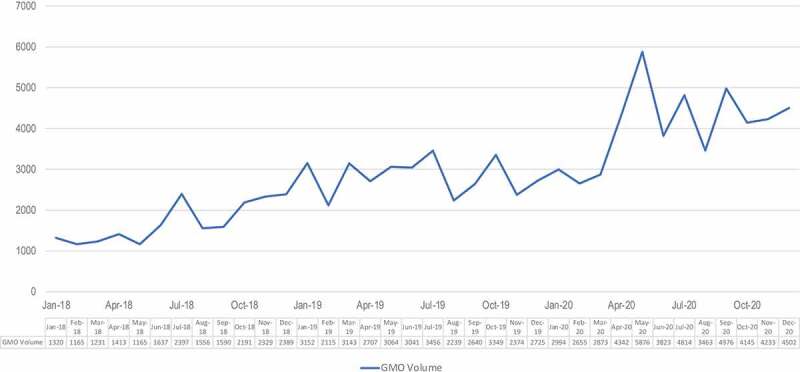
Volume of agricultural biotechnology GMO conversation in traditional media 2018–2020, showing the number of stories published.

As Fig. 1 shows, the volume of coverage of the GMO issue more than tripled in the time period we studied, from January 2018 (1320 articles) to December 2020 (4502 articles).

**Figure 2.**
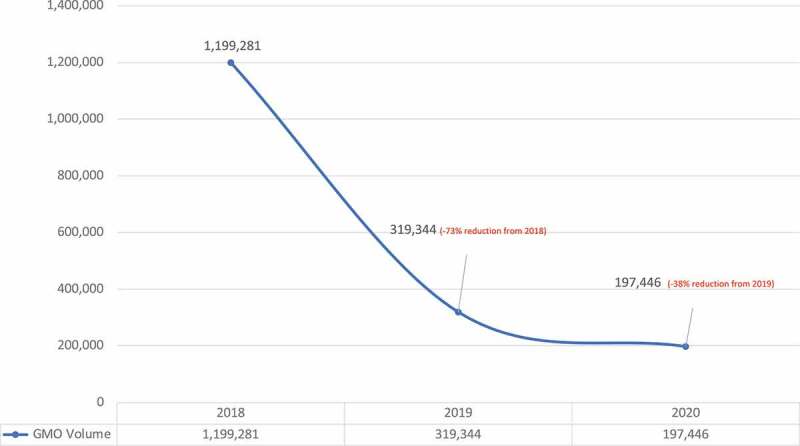
Volume of agricultural biotechnology social media interactions media 2018–2020.

The volume of social media interactions in the GMO conversation moved in the opposite direction however, showing a large decline between 2018 and 2020, falling from nearly 1.2 million to just under 200,000 in that time period, a decline of 82% (Fig. 2).

**Figure 3.**
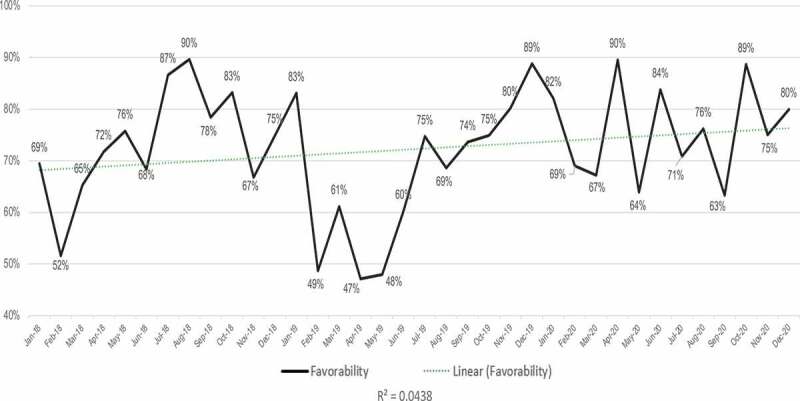
Sentiment analysis showing the favorability of the GMO conversation across all media (social and traditional combined) over a three-year period from Jan 2018 to Dec 2020.

The overall tone of the traditional and social media GMO conversation during the 2018 to 2020 period is generally favorable (Fig. 3). Favorability is defined as ‘positive’ and ‘neutral’ coverage as a percentage of the overall coverage, including ‘negative’ and ‘ambivalent’ coverage (see Methods). It is notable that the data are relatively noisy with high variance between the months in our sequence, ranging from a low of 47% in April 2019 to a high of 90% in April 2020. Overall favorability has increased somewhat over the three-year period, although the noisy data and relatively low R-squared value indicate low confidence in the robustness of this trend.

**Figure 4.**
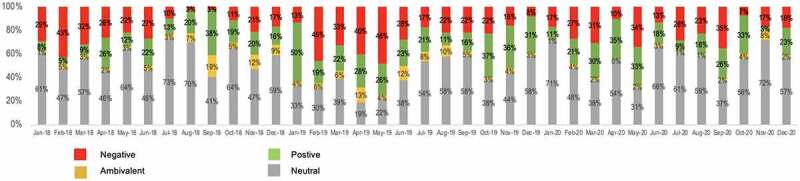
A monthly breakdown of sentiment across all media for the period Jan 2018 to Dec 2020.

The sentiment breakdown of the conversation on traditional and social media (combined) for the period of the study is depicted in Fig. 4. The data for Fig. 4 are the same as Fig. 3, with sentiment broken out into ‘negative,’ ‘positive,’ ‘ambivalent’ and ‘neutral’ categories rather than combined into a single overall favorability number for each month.

**Figure 5.**
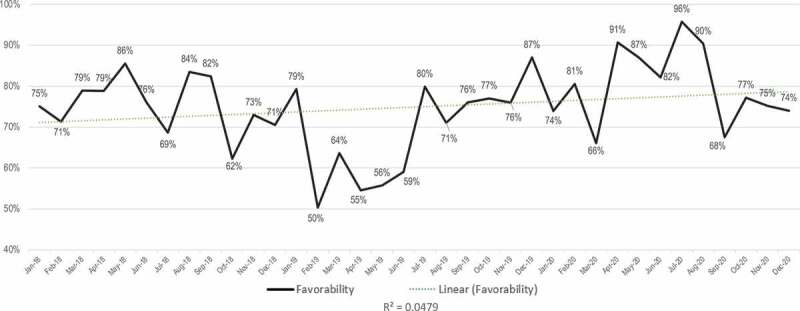
Traditional media sentiment analysis for the GMO conversation.

**Figure 6.**
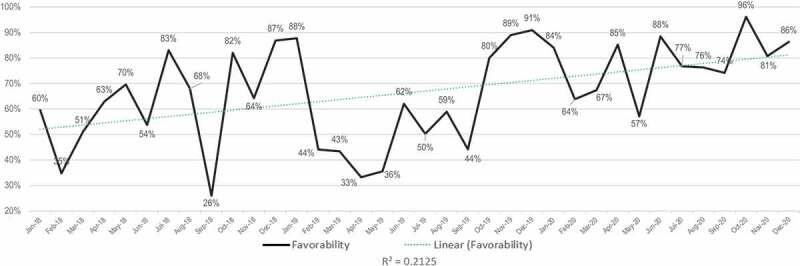
Social media sentiment analysis for the GMO conversation.

While Figs. 3 and 4 look at the favorability of all media with traditional and social combined, Figs. 5 and 6 deal with the sentiment of traditional and social media separately. The sentiment of the traditional media conversation around GMOs was slightly more positive than that of social media during the study period, averaging 75% favorable if neutral and overtly positive reporting are combined (Fig. 5) as compared with 67% favorability in social media (Fig. 6). Average monthly values as high as 96% favorable are found in traditional media, while throughout the whole period favorability never dropped below 50% (Fig. 5). However, as with the overall GMO conversation depicted in Figs. 3 and 5 shows noisy data with little confidence in the overall trend, with an R-squared value of 0.0479.

While sentiment toward GMOs in social media was substantially more variable than in traditional media, monthly values averaged in the 36-month time frame of the study show a strong long-term trend toward more positive social media coverage. While there were months in 2018 and 2019 when the favorability rating dropped to lows of 26% and 33%, it never dropped below 57% in 2020 (Fig. 5). Figure 5 appears to show a more robust linear trend toward greater favorability in social media than traditional media, with an R-squared value of 0.2125 accounting for 21% of the variance by time.

**Figure 7.**
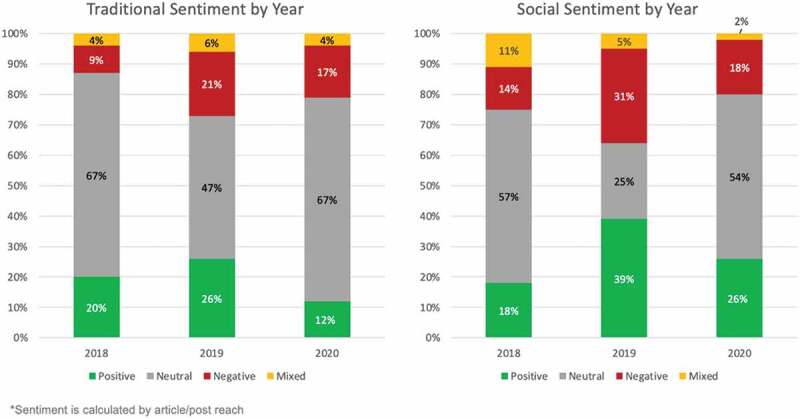
Average sentiment per year across traditional media and social media for 2018, 2019 and 2020.

Figure 7 shows annual averages of sentiment, broken into ‘positive,’ ‘negative,’ ‘neutral’ and ‘mixed’ categories for each year. As indicated above, one feature for 2018 and 2019 seems to be a substantially more negative sentiment seen in social media, although the two were almost equal in 2020.

**Figure 8.**
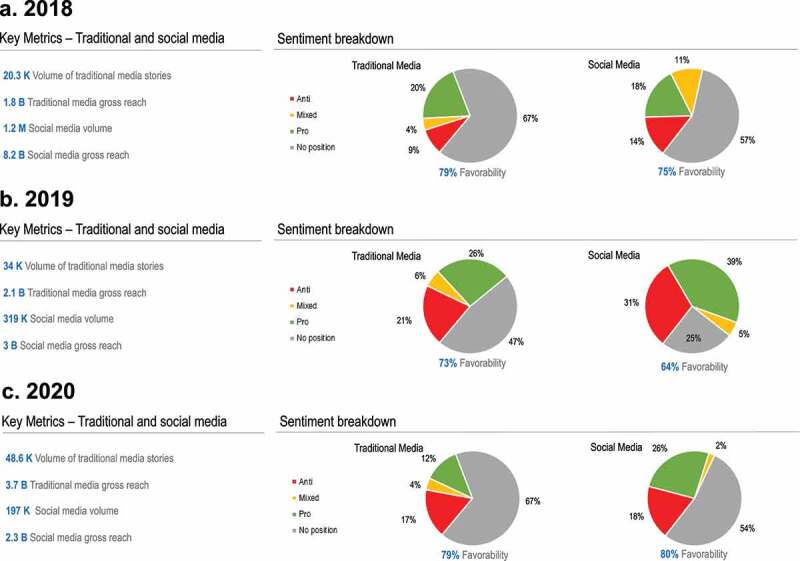
Key metrics for the GMO conversation in 2018 (a), 2019 (b) and 2020 (c), showing volume, gross reach and sentiment breakdown.

Figure 8 shows the key metrics for the GMO conversation. In terms of volume of content, there was an increase from 2018 to 2020, with 20,300 traditional media stories covering GMOs in 2018 (Fig. 8a) rising to 34,000 in 2019 (Fig. 8b) and 48,600 stories in 2020 (Fig. 8c). When assessed in terms of gross reach, the increase was from 1.8 billion to 3.7 billion over the same time period. There was a sharp downward trend in the visibility of the GMO issue on social media, however, from 1.2 million social posts in 2018 to 197,000 in 2020. This may suggest that despite an increase in ongoing traditional media coverage there is less salience in the GMO debate in the wider population as indicated in the sharp decline in the volume of social media posts, particularly when combined with the strong trend toward increased social media favorability seen in Fig. 6.

### The Monsanto/Bayer Effect

**Figure 9.**
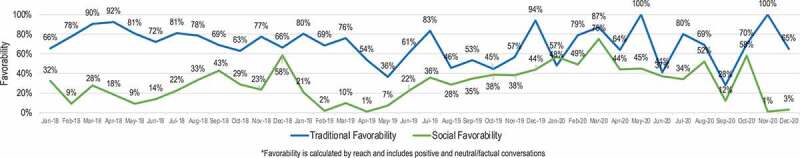
The favorability of the coverage of Monsanto/Bayer over the three-year period in traditional (blue) and social (green) media.

Monsanto (now part of Bayer) and its association with pesticides, notably glyphosate, appears to strongly drive negative perceptions toward GMOs. Coverage of Monsanto/Bayer in both traditional and social media was consistently and considerably more negative than coverage of GMOs overall. In some months almost the entirety of the social media conversation took a negative tone, such as April 2019 and November 2020, with only 1% favorability. (Fig. 9).

As with the general GMO issue, traditional media coverage of Monsanto/Bayer was substantially more favorable than social, reaching highs of 100% on occasion. About a quarter of the overall GMO debate involved mentions of glyphosate as an issue, whereas a third to nearly half of traditional media coverage of GMOs involved Monsanto/Bayer. References to glyphosate in social media declined by 3% over that period, while the figure is 4% for traditional media (Figure not shown).

#### Influence of Twitter Bots and Cyborgs

**Figure 10.**
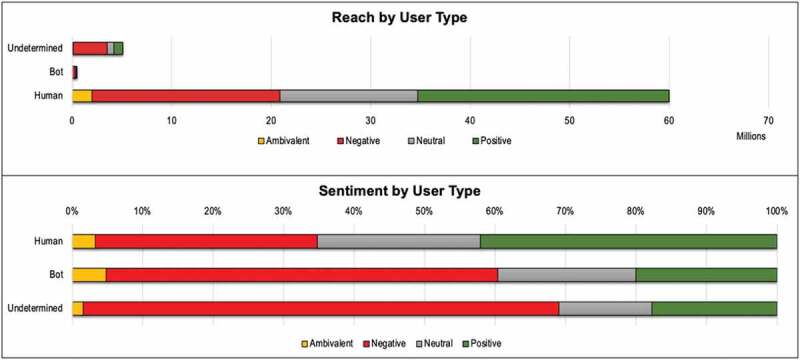
Role of Bots in GMO coverage 2018–2020.

Bot accounts represented 10% of Twitter users engaged in GMO discussions between 2018 and 2020 and contributed 10% of overall tweet volume. Bot accounts had much lower salience than human-operated accounts, contributing only 1% of gross reach. However, three out of the top ten Twitter accounts for volume of GMO content in 2019 were at least partially automated (listed as “undetermined” in Botometer scores) and so may appear to have influence due to the sheer volume of coverage (not shown). These cyborg accounts (human accounts that use automated posting for a large proportion of their content) were about 20% of overall accounts and were substantially more influential than bots. Combined, this suggests that about a third of users engaged in the GMO debate were cyborgs and bots. In addition, bots and cyborgs were substantially more negative in sentiment toward GMOs than human accounts. (Fig. 10)

#### GMOs in Africa and South Asia

The GMO conversation was different in Africa and South Asia than in the United States, which dominated in terms of overall volume and gross reach. The gross reach for the 2018 GMO conversation in the US was 3.6 billion, compared to 116 million in Kenya and 113 million in the Philippines, the two next largest geographies. It was just 2.6 million in Bangladesh (data not shown).

**Figure 11.**
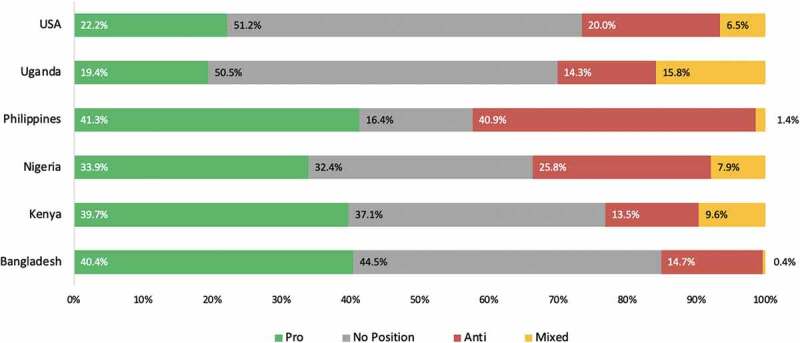
Sentiment analysis of GMO coverage (traditional and social media) in six geographies from 2018–2020.

In terms of sentiment analysis, though the conversation was generally favorable in all countries, it was more favorable in the US, with the Philippines registering the highest percentage of negative coverage (Fig. 11).

**Figure 12.**
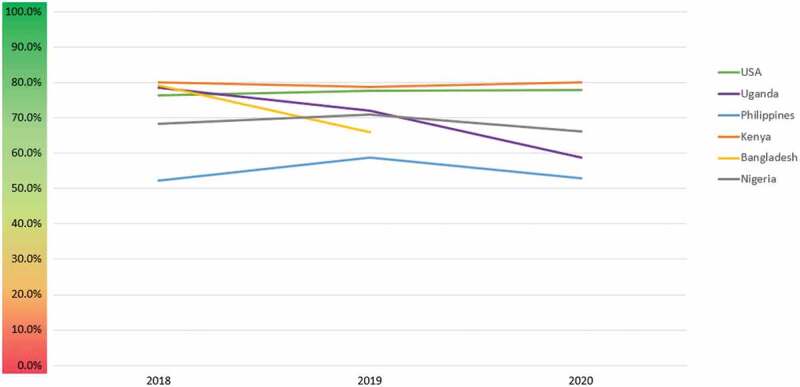
Favorability trends in six geographies.

In 2019, the average favorability increased over 2018, though there was a decline in some geographies in 2020. In the US and Kenya, the favorability remained relatively stable across the three years, whereas it dropped in Uganda and Bangladesh over time. In Nigeria and the Philippines, the favorability was greatest in 2019 (Fig. 12).

## Discussion

Although there has been substantial academic attention given to the course of the biotechnology debate in the media, previous assessments have typically been based on small data samples analyzed by hand, including at most a few hundred articles. We believe this analysis to be the first that attempts to portray a rough aggregate picture of the whole debate in the English language over a broad time period, using machine-learning tools to assess many thousands of articles with a potential reach of billions of combined views. To our knowledge it is also the first to include social media in this analysis and compare it w ith trends in traditional media over several years.

Previous studies have analyzed news reporting on GMOs, though often only for a small snapshot of time and without a comprehensive evaluation of media coverage. A 2010 paper, for example, analyzed six UK newspapers for the first three months of 2004, finding that scientists at the time were presented simply as one competing interest group with no special claim to truth.^10^ A study of Kenyan and international newspapers carrying biotechnology-related stories between 2010 and 2014 found that the publication of the 2012 Seralini study significantly increased the risk messaging in Kenyan reporting on the subject.^11^ Stephen Morse conducted an analysis of global newspaper reporting on genetically modified crops between 1996 and 2013, finding – perhaps surprisingly – mildly positive coverage during the period.^12^ Another long-term study, published more recently, looked at the Swedish GMO debate between 1994 and 2017.^13^ In volume terms, the number of articles rose to a broad peak in 2003–05, falling gradually until 2017. The researchers also found a clear trend from negative to positive during the period. Leonie Marks and colleagues, in a 2007 analysis of UK and US traditional media, found that coverage of biotechnology was markedly more positive for medical than agricultural applications.^14^

This type of analysis could be useful because high levels of skepticism about GM crops may be related to media coverage on the issue, which would thereby play an important role in shaping public opinion. In China, for instance, attitudes turned sharply negative following a 2012 scandal about a nutrition study involving genetically modified rice and Chinese children, which was brought to the fore by Greenpeace and widely reported with a narrative suggesting that genetically modified crops are instruments of Western control and imperialism.^15^ Prior to that, Chinese newspaper attitudes had been either positive or neutral toward GMOs.^16^ Media framing has also been strongly associated with a trend toward more negative public attitudes to GMOs in Russia in the years leading up to a ban imposed in 2016.^17^ These are not all recent trends: one study found that in Hungary, media framing of the GM issue largely favored the ‘anti’ side between 2007 and 2009.^18^

Media coverage of GMO issues does not arise in a vacuum. Instead, it reflects political, ideological, and economic contests in societies. In some cases, as in China, geopolitical anxieties can drive widespread public belief in conspiracy theories about Western aggression via genetic technologies. The Russian government, which is often accused of waging an information warfare campaign against the West, has also promoted fears and conspiracy theories about GMOs. A 2018 study found that the Russian state news networks RT and Sputnik produced many more articles on GMOs than Western media outlets, most of which were sharply negative.^19^ Some of these Russian-promoted stories featured conspiracy theories that were unlikely to gain exposure in conventional news, such as one headline in 2016: “GMO mosquitoes could be cause of Zika outbreak, critics say.”^20^

Negative coverage may also originate from groups ideologically opposed to genetic engineering, or NGOs that seek to raise campaign funds by spreading misinformation. This latter strategy has been termed the ‘monetization of disinformation’ and may raise millions of dollars per year for groups that employ this strategy as a fundraising tool. A recent study analyzing 95,000 online articles found that those receiving the most attention appeared not in conventional media but were published by “a small group of alternative health and pro-conspiracy sites.”^21^

Much of the controversy now takes place in the social media sphere, where trolls and bots can increase polarization and spread misinformation exponentially. A 2018 study of the vaccine issue found that trolls and bots often supported both sides in order to amplify controversy and create “false equivalency, eroding public consensus on vaccination.”^22^

Our analysis suggests that traditional media coverage of GMOs is consistently and substantially more neutral or positive than public perceptions as reported from polling data. This finding is in keeping with the media’s traditional role of aiming for neutral or impartial coverage. Because monthly favorability ratings rise and fall as different stories break, there is only a weak long-term trend toward more favorable coverage in traditional media seen in our data.

The situation is somewhat different on social media. In social media, extreme or one-sided positions can pass unchallenged and strong statements, regardless of whether they are true or false, tend to be ‘liked’ or shared more often. Yet even in this ‘free for all’ environment, monthly values averaged in the 36-month time frame of the study show a robust long-term trend toward more positive social media coverage.

In volume terms, there was a significant increase from 2018 to 2020 in traditional media coverage of the GMO issue. There was a sharp downward trend in the volume of GMO-related posts on social media, however. This suggests that the GMO issue is perhaps becoming somewhat less salient over time in terms of public engagement. This decline could however also be due in part to the COVID-19 pandemic, which may have occupied the attention of social media users during 2020. It also suggests that while traditional media coverage of the issue is typically driven by events happening in the news cycle, social media commentators are less driven by mainstream news coverage of the issue. It is notable that traditional and social media visibility peaks do not tend to occur at the same time, suggesting that the debates operate somewhat independently of each other.

A familiar factor in the GMO conversation is the antipathy directed specifically toward Monsanto, with the company becoming a bogeyman for anti-GMO activists and its flagship ‘RoundupReady’ crops coming to symbolize overall objections to the technology. Though Monsanto has since been purchased by Bayer and its name retired, the stigma seems to remain. We found that coverage of Monsanto/Bayer in both traditional and social media is consistently and considerably more negative than coverage of GMOs overall. This likely reflects ongoing negative portrayals of the company regarding pesticides and issues of corporate control of seeds, and thus food. In some months over the two-year period of January 2018 through December 2019, almost the entirety of the social media conversation took a negative tone, though favorable spikes were also recorded both years. The fact that the Monsanto/Bayer conversation was substantially more negative in terms of social media sentiment analysis than other areas helps validate our methods, as it confirms what might be expected given our broader understanding of the debate.

Geographically, the United States dominates the GMO conversation, both in terms of volume and reach. This may be because the technology is widely employed in US agriculture, which also has a robust presence in traditional and social media. The conversation is generally favorable in the US, Africa, and South Asia, though it remains divided in the Philippines, where GM corn has been adopted but international controversies remain over the recent adoption of GM Golden Rice. In Africa, the conversation is most negative in Uganda. These differences may be due to the fact that Nigeria and Kenya have recently adopted GM crops, with farmers and media seeing the positive results of field trials, while Uganda still lacks a biosafety law that would permit introduction of GM crops.

## Conclusion

Our analysis shows that traditional media tended to be somewhat more positive in their coverage than social media in 2018 and 2019, though that gap disappeared in 2020. While the volume of traditional media coverage of GMOs increased significantly during the period, this was combined with a dramatic drop in the volume of social media posts. Both traditional and social media saw trends toward increasing favorability, with the positive trend especially robust in social media.

Notably, the same positive favorability was observed in Africa, where countries are just beginning to adopt the technology. The favorable conversation in Kenya and Nigeria may be due to the fact that farmers have been able to witness field trials as well as plant GM seeds on their own farms. It may also be that anti-GMO activists lessen their activities in countries where the technology has been adopted, either turning to other issues or devoting their attention to countries that are still undecided.

Our analysis also found that cyborgs and bots represent about a third of the users engaged in the GMO social media debate. Furthermore, their posts are substantially more negative in sentiment toward GMOs than human accounts. This suggests that cyborgs and bots may be intentionally used by nefarious actors to sow dissent and make the GMO conversation appear more negative and polarized than it is.

The decline in volume of social media posts combined with a strong trend toward greater favorability may indicate a drop in the salience of the GMO debate among the wider population, even while the volume of coverage in traditional media increased. Overall, our results suggest that both social and traditional media may be moving toward a more favorable and less polarized conversation on ag biotech overall.

Despite these encouraging results, it is clear that the scientific community still faces major communications challenges in addressing gaps between traditional and social media debates and the actual scientific consensus around the safety and desirability of agricultural biotechnology. Although the situation appears to be improving, there is no guarantee that this will continue as the influence of negative sentiments and actors continues to weigh on the debate and skew public perceptions away from perspectives that are based on genuine scientific evidence.
